# Effects of resveratrol on apoptosis in a rat model of vascular dementia

**DOI:** 10.3892/etm.2014.1542

**Published:** 2014-02-13

**Authors:** ZHI-KUN SUN, XING-RONG MA, YAN-JIE JIA, YAN-RU LIU, JIE-WEN ZHANG, BO-AI ZHANG

**Affiliations:** 1 Department of Neurology, Henan Provincial People’s Hospital, Zhengzhou, Henan 450002, P.R. China; 2 Department of Neurology, The First Affiliated Hospital, Zhengzhou University, Zhengzhou, Henan 450002, P.R. China

**Keywords:** vascular dementia, resveratrol, apoptosis, caspase-3, poly(ADP-ribose) polymerase

## Abstract

Resveratrol is a natural polyphenol widely present in plants, particularly in the skin of red grapes and in wine. It possesses a wide range of biological effects and exhibits neuroprotective effects in numerous diseases. However, data evaluating the effects of resveratrol in vascular dementia (VaD) are lacking. In the present study, the permanent, bilateral common carotid artery occlusion rat model was used to study the effects of resveratrol on VaD. The Morris water maze was used to test the spatial learning and memory performance of the rats. The expression levels of Bax, Bcl-2, cleaved caspase-3 and cleaved poly(ADP-ribose) polymerase (PARP) in the hippocampus were measured. The results showed that resveratrol inhibited memory impairment in the VaD rat model, and attenuated the increases in the expression levels of Bax, cleaved caspase-3 and cleaved PARP and the reductions in the expression levels of Bcl-2 that were induced by VaD. These results provide a novel insight into the neuroprotective effects of resveratrol and its possible therapeutic role in VaD.

## Introduction

Apoptosis is a mode of cell death in which single cells are eliminated in the midst of living tissue. The term derives from a Greek word that is used for the dropping off of leaves from trees. It is characterized by structural changes that appear with marked fidelity in cells of widely different lineage, and presumably represent a pleiotropic effector response (1). Of the different mechanisms of cell death, apoptosis has been proposed to explain the cell loss observed in numerous neurological disorders, including Alzheimer’s disease, vascular dementia (VaD), Parkinson’s disease, Huntington’s disease, amyotrophic lateral sclerosis and stroke (2). VaD is the second most common type of dementia following Alzheimer’s disease. When the blood supply to the brain is reduced by a blocked or diseased vascular system, VaD occurs and leads to a progressive decline in memory and cognitive function (3). It is possible to induce chronic cerebral hypoperfusion by permanent bilateral occlusion of the common carotid arteries in rats, resulting in significant white matter lesions, learning and memory impairment (4), and hippocampal neuronal damage (5). Clinical evidence supports the hypothesis that chronic cerebral hypoperfusion is associated with cognitive decline in aging and in neurodegenerative disorders (6). Thus, permanent bilateral occlusion of the common carotid arteries in rats provides a model useful for understanding the pathophysiology of chronic cerebrovascular hypoperfusion and for screening drugs with potential therapeutic value for VaD (7).

Resveratrol, a polyphenolic compound in juice and wine, was first reported in 1939 by the Japanese researcher, Dr Michio Takaoka (8). It possesses a wide range of biological effects including anti-apoptotic (9,10), anti-oxidative (11), anti-inflammatory (12) and anti-carcinogenic properties (13). Resveratrol has been reported to exhibit neuroprotective effects in the models of numerous diseases, such as cerebral ischemia (14,15), kainic acid-induced excitotoxicity (16), Huntington’s disease (17), Parkinson’s disease (18) and Alzheimer’s disease (19). However, data evaluating the effects of resveratrol in VaD are lacking. In the present study, the permanent, bilateral common carotid artery occlusion rat model was used to attain information on the effects of resveratrol on VaD.

## Materials and methods

### Animals

Wistar rats (provided by the Henan Laboratory Animal Research Center, Zhengzhou, China), aged 12–14 months, weighing 300–400 g and of unlimited gender, were used in the study. The rats were housed in standard cages with liquid and food available *ad libitum*, at a mean ± standard deviation (SD) constant temperature of 22±2°C, humidity of 55±5% and under an artificial reversed 12-h light-dark cycle with the light off at 7.00 a.m. All procedures were conducted in accordance with the Guidance Suggestions for the Care and Use of Laboratory Animals, formulated by the Ministry of Science and Technology of the People’s Republic of China (20).

### Methods

#### Experimental design

The rats were randomly divided into four groups (n=20/group) as follows: Group A, normal control group in which the rats were subjected to sham surgery; group B, VaD model group in which the rats underwent VaD-modeling surgery; group C, resveratrol control group in which the rats were subjected to sham surgery and treated with resveratrol; group D, treatment group in which the rats underwent VaD-modeling surgery and were treated with resveratrol. The models of VaD were established by permanent bilateral occlusion of the common carotid arteries in groups B and D. The bilateral common carotid arteries were isolated but not ligated in groups A and C. The rats of groups C and D received a daily oral dose of 25 mg/kg resveratrol (obtained from Sigma-Aldrich, St. Louis, MO, USA) starting from 8 weeks after the surgery until 12 weeks after the surgery; and the rats of groups A and C received the same volume of ethanol. The study was approved by the Committee on Ethics of Life Sciences of the First Affiliated Hospital of Zhengzhou University (Zhengzhou, China).

#### Bilateral common carotid occlusion

The bilateral common carotid arteries of the rats were occluded as previously described by Ni *et al* (4). The rats were anesthetized with a 10% chloral hydrate (0.3 ml/100 g; Sigma-Aldrich) intraperitoneal injection. To prevent respiratory distress, the rats were also administered atropine sulfate (0.1 mg/kg, intramuscularly; Polfa Warszawa S.A., Warsaw, Poland). A midline incision was made to expose the bilateral common carotid arteries. The common carotid arteries were carefully separated from the surrounding tissues, including the vagus nerve, and ligated with Ethicon Coated Vicryl (polyglactin 910) plus antibacterial absorbable surgical suture (size 3-0; Johnson & Johnson Medical Ltd., Wokingham, UK), ~1 cm inferior to the origin of the external carotid artery. The control rats were subjected to the same surgical procedure without occlusion of the arteries.

#### Morris water maze test

The spatial learning and memory performance of the rats was measured using the Morris water maze task (provided by Chinese Academy of Medical Sciences, Beijing, China). The test was administered by an operator blinded to the group conditions. The Morris water maze consisted of a painted circular pool (120 cm in diameter and 30 cm in depth) in which the rats were trained to escape from the water by swimming to a hidden platform (9 cm in diameter) 1.5 cm beneath the surface, the location of which was only identifiable using distal extra-maze cues attached to the room walls. The water was maintained at 22°C and made opaque with titanium dioxide throughout all training and testing. The pool was divided into four quadrants: North (Target), south (Opposite), east (Adjacent 1) and west (Adjacent 2). The experiments were recorded using a camera connected to a video recorder and a computerized tracking system (Shanghai Jiliang Software Technology Co. Ltd., Shanghai, China).

The Morris water maze test procedure was conducted as previously reported (21). The first 4 days were the reference memory test phase, which consisted of 16 training trials: 4 training trials per day for 4 training days with an inter-trial interval of 30–40 min. At the beginning of each trial, the rat was placed into one of the four quadrants facing the wall. Although the starting point was randomly selected, the protocol was fixed at the beginning of each trial and was maintained throughout all trials. Each rat was allowed 180 sec to locate and mount the platform; 30 sec after the rat mounted the platform, it was removed, placed in a holding cage and warmed with a heat lamp. The rats that failed to locate and mount the platform within 180 sec were gently guided to the platform and required to remain there for 30 sec prior to being transferred to the holding cage. A video camera mounted above the pool was used to track the rats. The amount of time spent locating and mounting the platform (escape latency) and the swimming pathway prior to locating the platform (escape distance) were calculated from the recorded videos using Morris water maze software (Shanghai Jiliang Software Technology Co. Ltd., Shanghai, China). On the fifth day, a probe test was performed in which the platform was removed. The rats were placed into the water in one of the two quadrants adjacent to the platform (Adjacent 1 and Adjacent 2 quadrants) and were allowed to swim freely for 120 sec. The percentage of time spent and the swimming distance percentage in the target quadrant were recorded.

#### Collection and preservation of brain tissues

The rats were anesthetized with diethyl ether and then perfused with phosphate buffer saline (pH 7.4). The brain of each rat was immediately removed from the skull, and the hippocampus was dissected on ice. All brain tissues were stored at −80°C until biochemical analysis.

#### Western blot analysis

Western blot analysis was performed using the hippocampus of each rat, which had been dissected and stored at −80°C. The brain tissues were homogenized with lysis buffer [10 mM Tris pH 7.4, 100 mM NaCl, 1 mM ethylenediamine-N,N,N′, N′-tetraacetic acid (EDTA), 1 mM ethyleneglycol-bis(2-aminoethyl)-N,N,N′, N′-tetraacetic acid (EGTA), 1 mM NaF, 20 mM Na_4_P_2_O_7_, 2 mM Na_3_VO_4_, 0.1% sodium dodecyl sulfate (SDS), 0.5% sodium deoxycholate, 1% Triton-X 100, 10% glycerol, 1 mM phenylmethylsulfonyl fluoride (PMSF; made from a 0.3 M stock in dimethylsulfoxide), 60 μg/ml aprotinin, 10 μg/ml leupeptin, and 1 μg/ml pepstatin] for 30 min. The soluble fraction was obtained by centrifugation at 2,500 × g for 20 min at 4°C. The concentration of the protein was determined using a BCA assay (Pierce Biotechnology, Inc., Rockford, IL, USA). The western blotting procedure was conducted as previously reported (22). Equal amounts of protein (20 μg) were boiled at 100°C for 10 min in loading buffer (Fermentas, Beijing, China) and were separated in 8–10% SDS-polyacrylamide gel, and the resolved proteins were electrotransferred to polyvinylidene difluoride membranes (Bio-Rad, Hercules, CA, USA). Subsequently, the membranes were blocked with 5% non-fat milk in TBST (10 mM Tris-HCl pH 8.0, 150 mM NaCl and 0.2% Tween-20) for 1 h at room temperature and incubated with the appropriate primary antibody [1:200 Bax and Bcl-2; Santa Cruz Biotechnology, Inc., Santa Cruz, CA, USA; 1:5,000 β-actin, Sigma-Aldrich; and 1:1,000 cleaved caspase-3 and poly(ADP-ribose) polymerase (PARP), Cell Signaling Technology Inc., Beverly, MA, USA] at 4°C overnight. The membranes were then washed twice with TBST and probed with the corresponding secondary antibodies conjugated with horseradish peroxidase (HRP) (anti-mouse/rabbit-HRP was used at a dilution of 1:5,000) at room temperature for 1 h. The membranes were challenged with Vectastain ABC agent (Vector Laboratories, Burlingame, CA, USA). After washing, the signals were developed with a ECL Advance Western Blotting Detection kit (Amersham Pharmacia Biotech, Buckinghamshire, UK). The blots were stripped and reprobed with anti-β-actin as a loading control. The band intensities were quantified by densitometric analyses using an AxioCam digital camera and the KS400 photo analysis system, version 3.0 (Carl Zeiss, Oberkochen, Germany).

#### Statistical analysis

Data are expressed as the mean ± SD and were analyzed using SPSS statistical software, version 16.0 (SPSS, Inc., Chicago, IL, USA). Each procedure was performed in duplicate in three to five independent experiments. Statistical analyses were performed using one-way analysis of variance followed by two-tailed Student’s t-test, and statistical significance was assumed at P<0.05.

## Results

### Effects of resveratrol on memory impairment in VaD rat models

**Figure 1:**
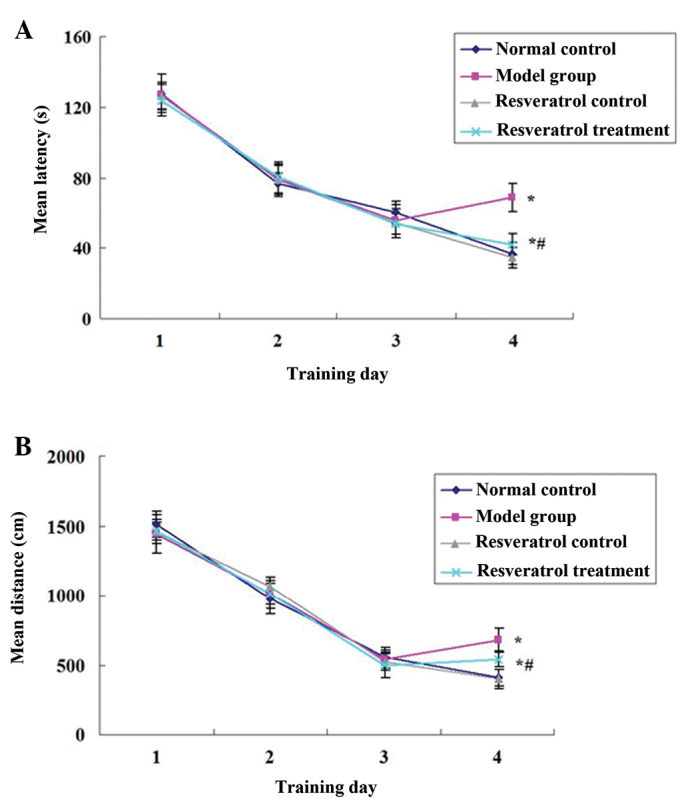
Effects of resveratrol on memory impairment in a VaD rat model. The Morris water maze was used to test the memory of the rats by measuring the escape latency and escape distance. Data are expressed as the mean ± SD. ^*^P<0.05, vs. the normal control group; ^#^P<0.05, vs. the model group. Statistical analyses were performed using one-way analysis of variance, followed by the two-tailed Student’s t-test. VaD, vascular dementia.

The Morris water maze was used to test the spatial learning and memory performance of the rats by measuring the escape latency (the amount of time spent locating and mounting the platform in the water maze) and escape distance (the swimming pathway prior to locating the platform in the water maze). In the first three days, no differences were identified in the escape latency (Fig. 1A; P>0.05) and escape distance (Fig. 1B; P>0.05) of any of the groups. On the fourth day, the escape latency and escape distance of the resveratrol control group were not significantly different compared with those of the normal control group (P>0.05), those of the model group were significantly higher than those of the normal control group (P<0.05), and all of the changes were partly attenuated by the resveratrol treatment. The escape latency and escape distance of the rats in the resveratrol treatment group were significantly shorter than those of the rats in the model group (P<0.05; Fig. 1).

**Figure 2:**
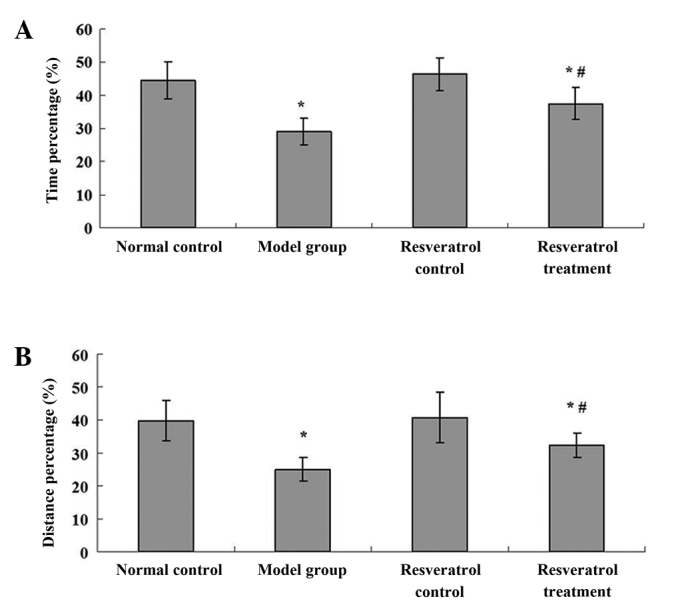
Effects of resveratrol on memory impairment in a VaD rat model. A probe test was perfomed to analyze maintenance of memory in a Morris water maze by measuring the percentage of (A) the time spent and (B) the swimming distance in the target quadrant. Data are expressed as the mean ± SD. ^*^P<0.05, vs. the normal control group; ^#^P<0.05, vs. the model group. Statistical analyses were performed using one-way analysis of variance, followed by the two-tailed Student’s t-test. VaD, vascular dementia.

Following the water maze training test, a probe test was performed to analyze the maintenance of memory, in which the platform was removed and the percentage of time spent and of the swimming distance in the target quadrant were recorded. During the probe test, the percentage of time spent (Fig. 2A) and of the swimming distance (Fig. 2B) in the target quadrant of the resveratrol control group were not significantly different compared with those of the normal control group (P>0.05), and those of the model group rats were significantly shorter than those of the normal control group rats (P<0.05), and the changes were partly attenuated by the resveratrol treatment. The percentage of time spent (Fig. 2A) and of the swimming distance (Fig. 2B) of the rats in the resveratrol treatment group were significantly longer than those of the rats in the model group (P<0.05; Fig. 2).

### Effects of resveratrol on Bax/Bcl-2 in the brains of VaD rat models

**Figure 3:**
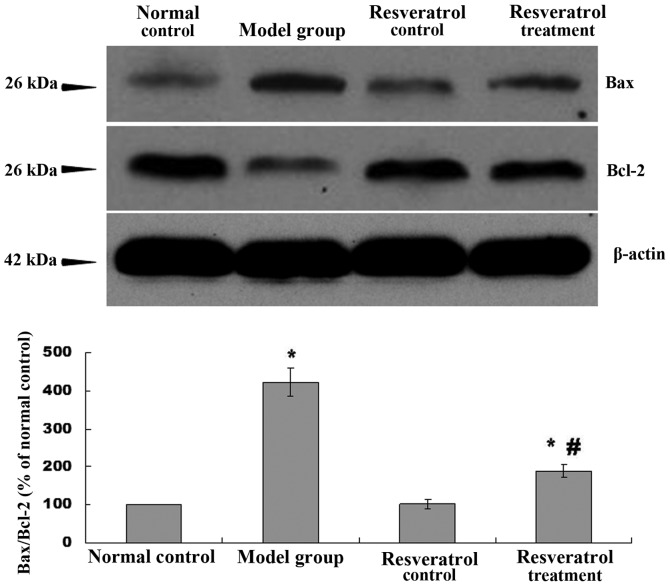
Effects of resveratrol on Bax/Bcl-2 in the brains of VaD rat models. Western blot analysis was used to measure the expression of Bax and Bcl-2. Three independent experiments were performed in duplicate. Data are expressed as the mean ± SD; the results are expressed as a ratio of the normal control group. ^*^P<0.05, vs. the normal control group; ^#^P<0.05, vs. the model group. Statistical analyses were performed using one-way analysis of variance, followed by the two-tailed Student’s t-test. VaD, vascular dementia.

Western blot analysis of the hippocampus lysates was performed using Bax and Bcl-2 antibodies. It was identified that the expression levels of Bcl-2 and Bax in the resveratrol control group were not significantly different compared with those of the normal control group (P>0.05). The expression levels of Bax were significantly increased, the expression levels of Bcl-2 were significantly reduced and the ratio of Bax/Bcl-2 was significantly increased in the model group rats compared with those of the normal control group (P<0.05). By contrast, the expression levels of Bax were significantly reduced, the expression levels of Bcl-2 were significantly increased and the Bax/Bcl-2 ratio was significantly decreased in the resveratrol treatment group rats compared with those of the model group rats (P<0.05; Fig. 3).

### Effects of resveratrol on the expression of cleaved caspase-3 in the brains of VaD rat models

**Figure 4:**
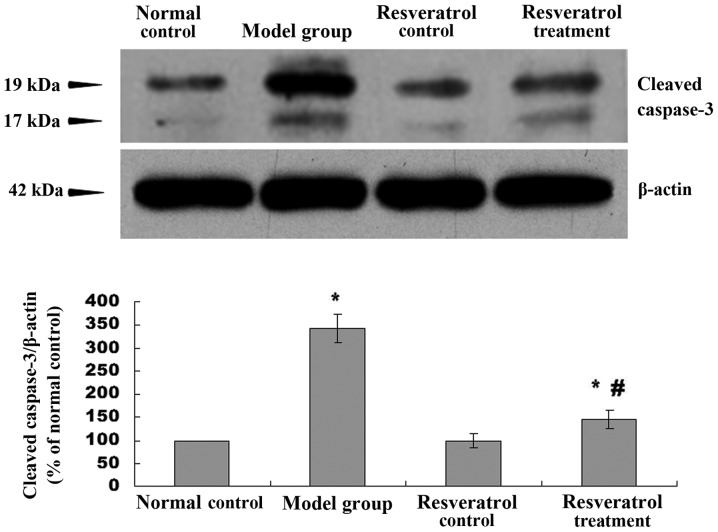
Effects of resveratrol on the expression of cleaved caspase-3 in the brains of VaD rat models. Western blot analysis was used to measure the expression of cleaved caspase-3. Three independent experiments were performed in duplicate. Data are expressed as the mean ± SD; the results are expressed as a ratio of the normal control group. ^*^P<0.05, vs. the normal control group; ^#^P<0.05, vs. the model group. Statistical analyses were performed using one-way analysis of variance, followed by the two-tailed Student’s t-test. VaD, vascular dementia.

Western blot analysis of the hippocampus lysates was performed using a cleaved caspase-3 antibody. It was identified that the expression of cleaved caspase-3 in the resveratrol control group was not significantly different compared with that of the normal control group (P>0.05), and the expression levels of cleaved caspase-3 were significantly increased in the model group rats compared with those of the normal control group rats (P<0.05). By contrast, the expression levels of cleaved caspase-3 were significantly reduced in the resveratrol treatment group rats compared with those of the model group rats (P<0.05; Fig. 4).

### Effects of resveratrol on the expression of cleaved PARP in the brains of VaD rat models

**Figure 5:**
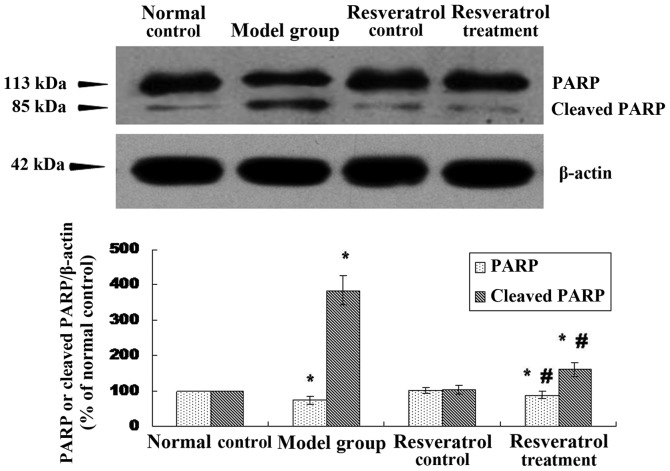
Effects of resveratrol on the expression of cleaved PARP in the brains of VaD rat models. Western blot analysis was used to measure the expression of cleaved PARP. Three independent experiments were performed in duplicate. Data are expressed as the mean ± SD; the results are expressed as a ratio of the normal control group. ^*^P<0.05, vs. the normal control group; ^#^P<0.05, vs. the model group. Statistical analyses were performed using one-way analysis of variance, followed by the two-tailed Student’s t-test. PARP, poly(ADP-ribose)polymerase; VaD, vascular dementia.

Western blot analysis of the hippocampus lysates was performed using a cleaved PARP antibody. It was identified that the expression of cleaved PARP in the resveratrol control group was not significantly different compared with that of the normal control group (P>0.05), and the expression levels of cleaved PARP were significantly increased in the model group rats compared with those of the normal control group rats (P<0.05). By contrast, the expression levels of cleaved PARP were significantly reduced in the resveratrol treatment group rats compared with those of the model group rats (P<0.05; Fig. 5).

## Discussion

Coinciding with population aging and improved survival from cardiovascular diseases and stroke, VaD is more frequent and is likely to affect an increasing number of patients in the future (23). Etiopathogenic mechanisms leading to VaD include oxidative stress, cytotoxicity of reactive oxygen species, mitochondrial dysfunction and apoptosis (24,25). Resveratrol has been proposed as a major constituent of the polyphenol fraction to which the health benefits of red wine consumption are attributed. *In vivo* and *in vitro* studies have shown that resveratrol exhibits neuroprotective effects in the models of numerous diseases. In the present study, the permanent, bilateral common carotid artery occlusion rat model was used to study the effects of resveratrol on VaD. Using a Morris water maze test, it was identified that the escape latency and escape distance (which are the time and distance travelled to reach the platform in the water maze) of the model group rats were significantly higher than those of the normal control group rats (P<0.05; Fig. 1). A probe test also showed that the percentage of time spent (Fig. 2A) and of the swimming distance (Fig. 2B) in the target quadrant of the model group rats were significantly shorter than those of the normal control group rats (P<0.05; Fig. 2). All of these changes were partly attenuated by the resveratrol treatment (P<0.05; Fig. 1 and 2) which suggests that resveratrol ameliorates the memory impairment in VaDmodel rats.

Apoptosis is a tightly regulated process, which involves changes in the expression of a distinct set of genes. Two of the major genes responsible for regulating apoptotic cell death are Bcl-2 and Bax. Bcl-2 is a key member of the anti-apoptotic Bcl-2 family that is crucial in regulating mitochondrial-mediated apoptotic cell death. Overexpression of Bcl-2 has been demonstrated to protect neuronal cells from neurotoxic insult (26). By contrast, Bax belongs to the pro-survival subfamily, which promotes apoptosis by translocation into the mitochondrial membrane and facilitating cytochrome *c* release, to propagate downstream apoptotic events (27). An elevated intracellular ratio of Bax to Bcl-2 occurs during increased apoptotic cell death (28,29). In the present study, it was demonstrated that the expression levels of Bax were significantly increased, the expression levels of Bcl-2 were significantly reduced and the ratio of Bax/Bcl-2 was significantly increased in the model group rats compared with those of the normal control group rats (P<0.05; Fig. 3). By contrast, the expression levels of Bax were significantly reduced, the expression levels of Bcl-2 were significantly increased and the ratio of Bax/Bcl-2 was significantly decreased in the resveratrol treatment group rats compared with those of the model group rats (P<0.05; Fig. 3).

Caspases are a family of cysteine proteases and are critical mediators of cell apoptosis. Caspases are important in the apoptotic process by two distinct pathways: The death receptor pathway and the mitochondrial pathway (30). Whichever pathway is involved, caspase-3 acts as an apoptotic executor. Caspase-3 activates DNA fragmentation factor, which in turn activates endonucleases to cleave nuclear DNA, and ultimately leads to cell death (31). Activation of caspase-3 appears to be a key event in the execution of the apoptotic cascade in numerous central nervous system diseases, such as Alzheimer’s disease and Down’s syndrome. In the present study, it was also identified that the expression levels of cleaved caspase-3 were significantly increased in the model group rats compared with those of the normal control group rats (P<0.05). By contrast, the expression levels of cleaved caspase-3 were significantly reduced in the resveratrol treatment group rats compared with those of the model group rats (P<0.05; Fig. 4).

Significant evidence indicates that caspase-3 is partially or totally responsible for the proteolytic cleavage of numerous key proteins, including PARP (32). PARP is a nuclear DNA-binding protein of 113 kDa that is constitutively expressed in eukaryotes and that comprises up to 1% of the total nuclear proteins (33). PARP is important for cell viability, and cleavage of PARP facilitates cellular disassembly and serves as a marker of cells undergoing apoptosis (34). In the present study, it was identified that the expression levels of cleaved PARP were significantly increased in the model group rats compared with those of the normal control group (P<0.05). By contrast, the expression levels of cleaved PARP were significantly reduced in the resveratrol treatment group rats compared with those of the model group rats (P<0.05; Fig. 5).

In conclusion, in the present study it was demonstrated that resveratrol was able to inhibit memory impairment in a VaD rat model. This effect was associated with attenuation of the increased expression levels of Bax, cleaved caspase-3 and cleaved PARP and decreased expression levels of Bcl-2 that were induced in the VaD model. These results confirm the neuroprotective effects of resveratrol on VaD and provide a novel insight into the neuroprotective effects of resveratrol and its possible therapeutic role in VaD.
